# Efficacy of intra-articular polynucleotides associated with hyaluronic acid vs hyaluronic acid alone in the treatment of knee osteoarthritis: A systematic review and meta-analysis of randomized clinical trial

**DOI:** 10.1097/MD.0000000000020689

**Published:** 2020-06-12

**Authors:** Lei Zhang, Ningbo Lei, Ruilong Chang, Chenxu Yang, Qiang Li, Ning Zuo, Yubiao Gu

**Affiliations:** The Third Department of Articular Bone, Gansu Provincial Hospital of Traditional Chinese Medicine, Lanzhou, China.

**Keywords:** hyaluronic acid, knee function, osteoarthritis, pain, polynucleotides

## Abstract

**Background:**

The reduced range of motion and pain are the most characteristic clinical features of osteoarthritis (OA). Hyaluronic acid (HA), which is one of the infiltrative therapies for OA treatment, and polynucleotides (PNs), which is a DNA-derived macromolecule favored cell growth and collagen production, are an ongoing debate in clinical effectiveness.

**Methods:**

We plan to perform a systematic review and meta-analysis of randomized clinical trial to evaluate efficacy of intra-articular polynucleotides associated with hyaluronic acid versus hyaluronic acid alone in the treatment of knee osteoarthritis. We will search PubMed, EMBASE, Cochrane Library using a comprehensive strategy. The related conference proceedings and reference lists of the included studies will also be checked to identify additional studies. Two reviewers will screen retrieved records, extract information and assess the risk of bias independently. Stata v15.1 software will be used to conduct data synthesis.

**Results:**

This study will be submitted to a peer-reviewed journal for publication.

**Conclusion:**

We hope it will provide a relatively comprehensive reference for clinical practice and future relevant clinical trials.

**Ethics and dissemination:**

Ethics approval and patient consent are not required, as this study is a systematic review and meta-analysis.

**PROSPERO registration number:**

CRD42020167678

## 1 Introduction

The degeneration of joint tissues and inflammatory microenvironment are primary characteristics of osteoarthritis (OA), which is the main reason of disability in older adults with decrement in work hours and daily life quality.^[1,2]^ With a prevalence that increases with age, OA is clinically characterized by pain and stiffness of entire joint and reduced range of motion, reportedly 10% patients older than 55 years has symptomatic radiographic knee.^[3]^

Current treatments consist of physiotherapy, changes in lifestyle, and intake of drugs included opioids, analgesics, corticoids, COX-2 inhibitors, and nonsteroidal anti-inflammatory drugs.^[4]^ Hyaluronic acid (HA), as the most commonly used infiltrative therapy, is a natural component of soft connective tissue with the ability to restore the viscoelastic properties of the synovial fluid and joint lubrication. It also has antiapoptotic, anti-inflammatory, antiangiogenic, and antifibrotic properties.^[5]^ Polynucleotides (PNs), a mixture of purines, pyrimidines, deoxyribonucleotides, and deoxyribonucleosides, links water and have viscoelastic property but also induce cell growth, collagen production, migration of several cell types, and can reduce inflammation.^[6–8]^ In preclinical and clinical studies PNs have shown positive results in musculoskeletal tissue regeneration^[9]^ and reduction in proteoglycan degradation and in metalloproteinase activity.^[10]^

Currently, the clinical effectiveness of PNs and HA are ongoing debate, and no meta-analysis evaluated the effects of the combination of PNs and HA in OA. To further confirm the efficacy of combination of PNs and HA [PNs associated with HA (PNHA)], we performed this systematic review and meta-analysis to explore the clinical outcomes of them.

## 2 Methods

### 2.1 Protocol registration

The protocol has been registered in PROSPERO, which is an International Prospective Register of Systematic Reviews. The registration number is CRD42020167678 (http://www.crd.york.ac.uk/PROSPERO/).^[11]^ The content of this protocol will follow the preferred reporting items for systematic review and meta-analysis protocols (PRISMA-P) recommendations.^[12]^ We also plan to conduct it in accordance with the Cochrane Handbook for the systematic reviews of interventions and preferred reporting items for systematic review and meta-analysis (PRISMA) guidelines.^[13]^

### 2.2 Eligibility criteria

#### 2.2.1 Types of studies

Randomized controlled trial (RCT) without published year, publication status limitations.

#### 2.2.2 Types of participants

Inclusion criteria required patients without pre-existing infiltrative therapies or patients with a single previous infiltration cycle, performed at least 6 months before enrollment. Exclusion criteria include pregnancy or breastfeeding, abuse of alcohol or drugs, systemic anticoagulants and steroids ongoing or suspended for less than 1 month, hematological diseases or local skin lesions in the site of treatment inoculation, patients with pre-existing infiltrative therapies or patients with a single previous HA infiltration cycle, performed less than 6 month before enrollment and Hypersensitivity to the therapeutic products, previous bone fractures, knee severe trauma, joint deformities, rheumatoid arthritis, articular inflammatory diseases, and previous surgery.

#### 2.2.3 Types of interventions and comparators

(1) the study group, treated with intra-articular injection of PNHA and
(2) the control group, treated with intra-articular injection of HA.

#### 2.2.4 Types of outcome measures

Outcomes were mainly identified by relevant literature and existed clinical practice. The evaluations of clinical function and pain were performed with the WOMAC score^[14]^ and knee society score^[15]^ at 0 and after 2, 6, and 12 months from the beginning of the treatments. Besides, all the endpoints reported in the included studies will be collected and evaluated, although we may not mention some of them in this protocol.

### 2.3 Literature search

A systematic search of the literature will be conducted without language and year restrictions to identify all relevant RCT. We will search following electronic databases: PubMed, EMBASE and Cochrane Library from 2002 to May 2020 using related search terms, including “hyaluronic acid”, “knee osteoarthritides,” “osteoarth.” In addition, congress and conference proceedings will be manually retrieved. Related articles and references of included research will also be tracked to find potential studies. If significant data was incomplete in included study, we will contact the authors to get unpublished data.

### 2.4 Study selection and data extraction

After imported into the Endnote X7 and duplication, retrieved records will be independently screened by 2 reviewers (LZ and NBL). First, we will read the titles and abstracts of all indentified records to exclude clearly unrelated records based on the inclusion criteria. Then the full texts of the articles retained were reviewed to further determine their suitability. Any disagreement will be resolved by a third reviewer (YBG or NZ). We will show the selection process in details in the PRISMA flow chart.

Two authors (RLC and CXY) of this review will independently extract the data using a pre-defined form. The basic characteristics, related outcome and quality evaluation information of included studies will be collected. Similarly, any discrepancies will be resolved by a third reviewer (YBG or NZ). Data extracted will include author, year, study type, number of participants, intervention, control, demographics, complications, previous history, the follow-up time and WOMAC score and knee society score.

### 2.5 Quality of evidence assessment

The quality of included studies will be assessed by grading of recommendations assessment development and evaluation (GRADE), and divided into 4 levels: high quality, moderate quality, low quality, and very low quality.^[16]^

### 2.6 Assessment of study bias

Included study bias will be independently assessed by 2 reviewers (LZ and QL) and any disagreement will be solved by a third reviewer (YBG or NZ). For randomized controlled trials, we will use the Cochrane risk of bias tools to evaluate potential bias in 7 specific domains:

(1) sequence generation,
(2) allocation concealment,
(3) blinding of participants and personnel,
(4) blinding of outcome assessment,
(5) incomplete outcome data,
(6) selective outcome reporting,
(7) other bias.^[17]^

### 2.7 Statistical analysis

For dichotomous variables, The Relative Risk with 95% confidence intervals (CI) were calculated from each study. Continuous variables will be presented as Standard Mean Difference with 95% CI. All endpoints will be combined and performed meta-analysis by using DerSimonian and Laird random effects model.^[18]^ We assessed statistical heterogeneity by using Chi^2^ test and I^2^ statistic. We will consider significant heterogeneity when *P* < .10 for Chi^2^ or I^2^ > 50%.^[19]^ All primary analyses were performed with STATA v15.1 (Stata Corp, College Station, TX).

#### 2.7.1 Subgroup analysis

We will also conduct subgroup analysis to find more potential information based on pre-set criteria in 4 variables:

(1) different patient age,
(2) different follow-up time.

#### 2.7.2 Sensitivity analysis

If the heterogeneity is high, we will conduct sensitivity analyses based on the patient age and follow-up time.

#### 2.7.3 Publication bias

The likelihood of publication bias was assessed graphically through the generation of funnel plots, evaluated using an Egger test.^[20]^

## 3 Results

The study does not require ethical approval because the meta-analysis is based on published research and the original data are anonymous. And this study will eventually be published in a peer-reviewed journal in the form of a scientific paper.

## 4 Discussion

To our knowledge, this is the first systematic review and meta-analysis concerning the efficacy of combination of PNs and HA [PNs associated with HA (PNHA)]. The results from our research may provide meaningful evidence for clinical practice and give a valuable reference for future study.

There seem to be some potential limitations for our study. First, we only include English language articles, which might miss some important data in other language article. In addition, only RCT and no cohort studies will be included in our study, which may have an obstacle to our data pooling and results interpretation. But it probably help to promotes several more reliable conclusions and focus on more precious direction for future clinical studies to some extent. Notwithstanding its limitation, we hope to provide a prompt and credible evaluation for efficacy of intra-articular PNHA vs HA alone in the treatment of knee osteoarthritis.

## Author contributions

Contributions LZ and NBL conceived the idea for this study; LZ and RLC designed the meta-analysis; CXY and QL provided statistical advice and input; LZ and NBL drafted the protocol; YBG and NZ reviewed the protocol and provided critical feedback.

## References

[1] Veronesi F Giavaresi G Maglio M Chondroprotective activity of Nacetyl phenylalanine glucosamine derivative on knee joint structure and inflammation in a murine model of osteoarthritis. Osteoarthr Cartil 2017;25:589–99. 27836674 10.1016/j.joca.2016.10.021

[2] Liu-Bryan R Terkeltaub R Emerging regulators of the inflammatory process in osteoarthritis. Nat Rev Rheumatol 2015;11:35–44. 25266449 10.1038/nrrheum.2014.162 PMC4374654

[3] Loeser RF Goldring SR Scanzello CR Osteoarthritis: a disease of the joint as an organ. Arthritis Rheum 2012;64:1697–707. 22392533 10.1002/art.34453 PMC3366018

[4] Gallagher B Tjoumakaris FP Harwood MI Chondroprotection and the prevention of osteoarthritis progression of the knee: a systematic review of treatment agents. Am J Sports Med 2015;43:734–44. 24866892 10.1177/0363546514533777

[5] Migliore A Procopio S Effectiveness and utility of hyaluronic acid in osteoarthritis. Clin Cases Miner Bone Metab 2015;12:31–3. 10.11138/ccmbm/2015.12.1.031 PMC4469223 26136793

[6] Bitto A Polito F Irrera N Polydeoxyribonucleotide reduces cytokine production and the severity of collagen-induced arthritis by stimulation of adenosine A(2A) receptor. Arthritis Rheum 2011;63:3364–71. 21769841 10.1002/art.30538

[7] Chung KI Kim HK Kim WS The effects of polydeoxyribonucleotide on the survival of random pattern skin flap in rats. Arch Plast Surg 2013;40:181–6. 23730590 10.5999/aps.2013.40.3.181 PMC3665858

[8] Kim SK Huh CK Lee JH Histologic study of bone-forming capacity on polydeoxyribonucleotide combined with demineralized dentin matrix. Maxillofac Plast Reconstr Surg 2016;38:7. 26913276 10.1186/s40902-016-0053-5 PMC4752573

[9] Veronesi F Dallari D Sabbioni G Polydeoxyribonucleotides (PDRNs) from skin to musculoskeletal tissue regeneration via adenosine A2A receptor involvement: a mini-review. J Cell Physiol 2017;232:2299–307. 27791262 10.1002/jcp.25663

[10] Gennero L Denysenko T Calisti GF Protective effects of polydeoxyribonucleotides on cartilage degradation in experimental cultures. Cell Biochem Funct 2013;31:214–27. 23001693 10.1002/cbf.2875

[11] Ge L Tian JH Li YN Association between prospective registration and overall reporting and methodological quality of systematic reviews: a meta-epidemiological study. J Clin Epidemiol 2018;93:45–55. 29111471 10.1016/j.jclinepi.2017.10.012

[12] Shamseer L Moher D Clarke M Preferred reporting items for systematic review and meta-analysis protocols (PRISMA-P) 2015: elaboration and explanation. BMJ 2015;350:g7647. 25555855 10.1136/bmj.g7647

[13] Moher D Liberati A Tetzlaff J Preferred reporting items for systematic reviews and meta-analyses: the PRISMA statement. PLoS Med 2009;6:e1000097. 19621072 10.1371/journal.pmed.1000097 PMC2707599

[14] Bellamy N Buchanan WW Goldsmith CH Validation Study of WOMAC: a health status instrument for measuring clinically-important-patient-relevant outcomes following total hip or knee arthroplasty in osteoarthritis. J Orthop Rheumatol 1988;1:95–108. 3068365

[15] Insall JN Dorr LD Scott RD Rationale of the Knee Society clinical rating system. Clin Orthop Relat Res 1989;248:13–4. 2805470

[16] Puhan MA Schunemann HJ Murad MH A GRADE Working Group approach for rating the quality of treatment effect estimates from network meta-analysis. BMJ 2014;349:g5630. 25252733 10.1136/bmj.g5630

[17] Corbett MS Higgins JP Woolacott NF Assessing baseline imbalance in randomised trials: implications for the Cochrane risk of bias tool. Res Synth Methods 2014;5:79–85. 26054027 10.1002/jrsm.1090

[18] DerSimonian R Laird N Meta-analysis in clinical trials. Control Clin Trials 1986;7:177–88. 3802833 10.1016/0197-2456(86)90046-2

[19] Higgins JP Thompson SG Deeks JJ Measuring inconsistency in meta-analyses. BMJ 2003;327:557–60. 12958120 10.1136/bmj.327.7414.557 PMC192859

[20] Egger M Davey Smith G Schneider M Bias in meta-analysis detected by a simple, graphical test. BMJ 1997;315:629–34. 9310563 10.1136/bmj.315.7109.629 PMC2127453

